# Extremely Elevated Creatine Kinase in COVID-19-Associated Rhabdomyolysis

**DOI:** 10.7759/cureus.45448

**Published:** 2023-09-18

**Authors:** Rachel E Laches, Sophie Tillotson, Erin Kaufman, Mohammad As Sayaideh

**Affiliations:** 1 Internal Medicine, University of Florida, Gainesville, USA

**Keywords:** clinical nephrology, viral infection, creatine kinase, covid 19, non-traumatic rhabdomyolysis

## Abstract

Rhabdomyolysis is a condition characterized by the destruction of skeletal muscle cells with the release of myoglobin and creatine kinase into the blood. Viral infections such as influenza and Severe acute respiratory syndrome coronavirus 2 (SARS‑CoV‑2) have been associated with rhabdomyolysis with varying degrees of morbidity and mortality. We present the case of a male in his early thirties who was diagnosed with rhabdomyolysis associated with coronavirus disease 2019 (COVID-19) infection who developed excessively high creatine kinase levels, peaking at 1,650,000 U/L. He was treated with IV fluids and made a complete recovery.

## Introduction

Rhabdomyolysis is a condition of rapid skeletal muscle destruction wherein skeletal sarcolemma rupture, resulting in extensive release of intracellular components into the bloodstream. Creatine kinase (CK), myoglobin, lactate dehydrogenase, and many other proteins and electrolytes are released into systemic circulation upon necrosis of skeletal muscle [1]. Myoglobin is freely filtered by the glomerulus, and the resulting myoglobinuria puts patients at risk for acute kidney injury and is of prominent concern to physicians treating patients with rhabdomyolysis as acute renal failure is a life-threatening condition [1-3].

The etiologies of rhabdomyolysis are varied and include, but are not limited to, trauma, exertion, infection, and genetic, endocrine, and autoimmune disorders [1]. Many bacteria, viruses, fungi, and protozoa have been identified as potential causative agents of rhabdomyolysis, though the mechanism of injury is less clear [2]. Muscle damage by viruses, such as coronavirus disease 2019 (COVID-19), could potentially be caused by direct muscle invasion by virus particles or potentially due to a large immune system response to the infection itself [4]. 

Viral infection is a recognized cause of rhabdomyolysis, which often initially presents with myalgias, as many viruses also do without concurrent rhabdomyolysis [1]. Numerous case studies have specifically reported associations between COVID-19 infection and rhabdomyolysis [4-14]. Thus, it is important to monitor COVID-19 patients with appropriate physical exams and labs in order to diagnose rhabdomyolysis early, initiate treatment, and mitigate downstream effects of proteinemia and myoglobinuria, such as acute kidney injury [1]. We present a patient with a past medical history of rhabdomyolysis following influenza infection who presented to the emergency department with COVID-19 infection and subsequent rhabdomyolysis with CK levels rising to 1600 times the upper threshold of normal during admission. 

## Case presentation

A male patient in his early thirties working as a personal trainer with a past medical history significant for rhabdomyolysis after a previous influenza infection presented to a stand-alone emergency department with a one-day history of fevers (T_max_ 38.9^o^C) and was diagnosed with COVID-19 infection and discharged. He returned to the emergency department the next day with muscle aches and dark urine. His CK at this time was 2910 U/L, his creatinine was 1.16 mg/dL, and his blood urea nitrogen (BUN) was 19 mg/dL. He declined admission to the hospital and was discharged home with instructions to drink fluids. He returned to the emergency department three days later with decreased urine output and dark urine. His CK at this time was over 40,000 U/L. His physical exam was significant only for mild bibasilar crackles and mild lower extremity muscle tenderness. He was diagnosed with rhabdomyolysis associated with COVID-19 infection and admitted to the hospital for intravenous fluids, initially at 200 cc/hour, and monitoring of kidney function. 

The patient’s CK continued to climb throughout admission, but his muscle tenderness and urine output improved. Nephrology was consulted on day three of admission when his CK continued to climb and recommended increasing his IV fluid rate to 400 cc/hour. His CK peaked on day four of admission at 1,615,500 U/L, but his creatinine and BUN remained within normal limits. By day five of admission, his CK had dropped to 164,400 U/L, and by day six further decreased to 73,000 U/L. His IV fluids were decreased to 300 cc/hour and his urine became clear. On the day of discharge, his CK had dropped to 37,311 U/L and he had complete resolution of symptoms. 

**Figure 1:**
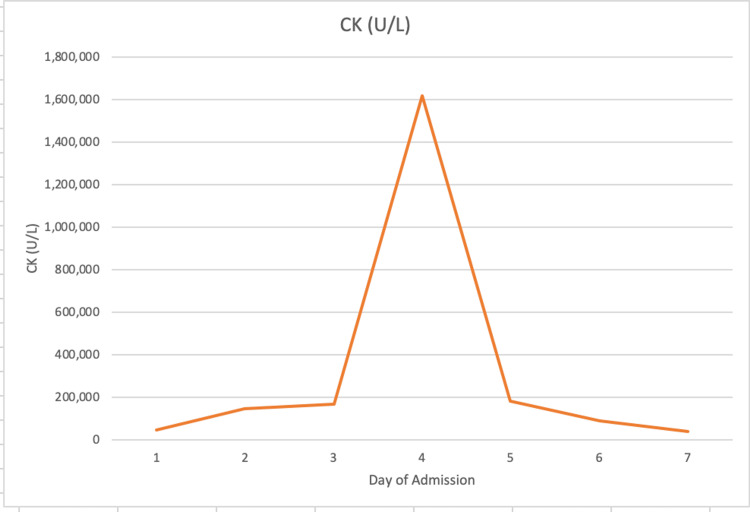
The patient's creatine kinase (CK) lab values trending over days of admission, rising sharply from 166,610 (U/L) on day 3 to peak at 1,615,500 (U/L) on day 4 before returning to 179,800 (U/L) on day 5

The patient made a complete recovery with no need for hemodialysis during his illness. He was able to return to his normal activities and return to work with no complications. Table 1 and Figure 1 depict his relevant laboratory values throughout his admission. After discharge, the patient underwent genetic testing to identify any metabolic or mitochondrial myopathies and was found to have a heterozygous mutation of the *PGAM2 *gene, which encodes the enzyme phosphoglycerate mutase. Homozygous mutation of *PGAM2 *is associated with glycogen storage disease type X, which is characterized by exercise intolerance, cramps [15], elevated CK, and myoglobinuria [16]. The patient was not diagnosed with glycogen storage disease type X or any other metabolic myopathy; he declined muscle biopsy and has remained asymptomatic even though he regularly engages in intense exercise. 

**Table 1:** Patient lab values throughout admission

Day of Admission	Creatine Kinase (U/L)	Creatinine (mg/dL)	Blood Urea Nitrogen (mg/dL)
1	>40,000	1.26	22
2	140,040	1.09	11
3	166,610	1.04	11
4	1,615,500	0.97	11
5	179,800	0.84	10
6	87,990	0.90	10
7	37,311	0.90	10

## Discussion

Rhabdomyolysis is a condition characterized by skeletal muscle necrosis and the release of intracellular proteins such as myoglobin, CK, and lactate dehydrogenase into the bloodstream [1,2]. Possible causes include traumatic muscular damage or overexertion as well as non-traumatic causes including infections, drug ingestion, metabolic diseases, and electrolyte disorders [2]. Rhabdomyolysis often presents with dark urine, myoglobinuria, and muscle aches, although more than half of patients do not experience muscular symptoms [1]. Elevated CK is the diagnostic hallmark of rhabdomyolysis, but electrolyte imbalances such as hyperkalemia and hyperphosphatemia may also be seen [1]. Rhabdomyolysis can result in acute kidney injury (AKI), so monitoring of BUN/creatinine ratio is important for early detection and treatment [1,2]. 

Regardless of the etiology of rhabdomyolysis, the pathophysiology follows the same cascade. Skeletal muscle injury damages the sarcolemma and depletes adenosine triphosphate (ATP), resulting in pathologically elevated intracellular calcium. This promotes cell lysis and the release of intracellular components into the bloodstream, causing further pathology in other organs, such as the kidneys, as myoglobin is freely filtered by the glomerulus and enters the renal tubules [3]. AKI in rhabdomyolysis is believed to be caused by the myoglobinuria present, though the exact mechanism of injury is unclear [3]. 

The various causes of rhabdomyolysis have fairly consistent clinical presentations across etiologies with symptomatic presentation directly related to muscle necrosis in addition to symptoms from secondary injuries, like AKI. The three most common symptoms patients present with are myalgias, muscle weakness, and tea-colored urine. While these are the most common symptoms, the triad appears in less than 10% of patients and diagnosis can be made without this classic presentation, especially when risk factors, such as trauma, immobilization, infection, and known muscular injury are present [1]. The gold standard laboratory value for diagnosing rhabdomyolysis is an elevated CK level, as this is released from damaged skeletal muscle cells. Though elevated myoglobin, not CK, is the direct cause of tissue damage itself, CK has a significantly longer half-life than myoglobin, so CK laboratory values are preferred for diagnosis [1]. Thus, diagnosis is typically made when CK laboratory values are five times the upper threshold of normal, 1000 U/L, as this concentration indicates a much higher risk for a patient to develop acute kidney injury. 

Treatment for rhabdomyolysis is focused on managing the underlying cause of muscle damage and preventing serious complications, such as acute kidney injury [1,2]. For drug-induced rhabdomyolysis, treatment includes medication cessation and patient education; for rhabdomyolysis caused by infection, treatment involves antibiotics, antivirals, or other infection management. Concurrently, physicians attempt to prevent AKI, a significant complication of rhabdomyolysis, through early and aggressive hydration using intravenous fluids. By increasing blood volume through fluid replacement, the kidneys have increased perfusion, preventing ischemia and further damage [1,2]. When treating rhabdomyolysis, fluids should be given at a level that maintains 300 mL urine an hour for the first 24 hours of treatment; they can then be titrated to maintain 200-300 mL urine an hour for the remainder of treatment until CK levels decline, renal function improves, and the patient stabilizes [2]. 

Viral infection is a recognized cause of rhabdomyolysis which often initially presents with myalgias. Similar cases of rhabdomyolysis associated with COVID-19 infection have been reported in the literature beginning in the early months of the pandemic [4-13]. Our report demonstrates a case of rhabdomyolysis with an extremely high CK value that was treated with a high flow rate of IV fluids and resolved completely with no residual kidney disease and no need for hemodialysis during treatment. Cases with comparable CK levels have been compiled in Table 2. While many of the cases were resolved with appropriate treatment, some of the patients required outpatient hemodialysis or ultimately passed away from the disease.

**Table 2:** Cases of rhabdomyolysis associated with COVID-19 infection with extremely high CK levels CK: creatine kinase; Cr: creatinine; COVID-19: coronavirus disease 2019; M: male; F: female

Author	Age	Gender	Peak CK (U/L)	Peak Cr (mg/dL)	Outcome
Chedid et al., 2020 [4]	51	M	464,000	19.09	Discharged to outpatient hemodialysis
Patel et al., 2021 [5]	60	M	685,000	5.88	Deceased
Anklesaria et al., 2020 [6]	57	M	923,120	7.6	Deceased
Shanbhag et al., 2020 [7]	19	M	694,200	0.982	Discharged
Kar et al., 2022 [8]	50	F	447,800	1.3	Deceased
Chetram et al., 2021 [9]	62	M	327,629	10	Discharged
Egoryan et al., 2021 [10]	50	M	359,910	3.51	Discharged
Fadel et al., 2021 [11]	33	M	362,445	1.0	Discharged
Li et al., 2021 [12]	22	F	764,470	8.13	Discharged
Yu et al., 2022 [13]	19	M	346,695	1.82	Discharged

## Conclusions

Viral infections, including COVID-19, are one of the most common causes of rhabdomyolysis. Early recognition and management of rhabdomyolysis are crucial to preserve renal function and avoid dialysis. In cases of severe rhabdomyolysis, the early involvement of nephrology is crucial for optimal treatment outcomes. Patients with significantly elevated CK levels should be referred to medical geneticists for an extensive evaluation of metabolic and mitochondrial myopathies.
